# Efficiency enhancement of non-selenized Cu(In,Ga)Se_2_ solar cells employing scalable low-cost antireflective coating

**DOI:** 10.1186/1556-276X-9-331

**Published:** 2014-07-04

**Authors:** Bao-Tang Jheng, Po-Tsun Liu, Meng-Chyi Wu

**Affiliations:** 1 Department of Electrical Engineering, National Tsing Hua University, Hsinchu City 30013, Taiwan; 2 Department of Photonics & Display Institute, National Chiao Tung University, Hsinchu City 30010, Taiwan

**Keywords:** Thin-film solar cell (TFSC), Zinc oxide (ZnO), Anti-reflection (AR) coating

## Abstract

In this study, a non-selenized CuInGaSe_2_ (CIGS) solar device with textured zinc oxide (ZnO) antireflection coatings was studied. The ZnO nanostructure was fabricated by a low-temperature aqueous solution deposition method. With controlling the morphology of the solution-grown tapered ZnO nanorod coatings, the average reflectance of the CIGS solar device decreased from 8.6% to 2.1%, and the energy conversion efficiency increased from 9.1% to 11.1%. The performance improvement in the CuInGaSe_2_ thin-film solar cell was well explained due to the gradual increase of the refractive index between air and the top electrode of solar cell device by the insertion of the ZnO nanostructure. The results demonstrate a potential application of the ZnO nanostructure array for efficient solar device technology.

## Background

Antireflection coatings play a major role in enhancing the efficiency of photovoltaic devices by increasing light coupling into the region of the absorber layers. Presently, the standard antireflection coatings in thin-film solar cells are the transparent thin films with quarter-wavelength thickness. In addition, the quarter-wavelength thickness antireflection coating is typically designed to suppress optical reflection in a specific range of wavelengths [1,2]. Also, it works only in a limited spectral range for a specific angle of incidence, typically for near-normal incidence. Recently, the availability of nanofabrication technology has enabled the engineering of materials with desired antireflection characteristics such as electron beam lithography and dry etching, which have been widely used to fabricate different antireflection nanostructures [3,4]. However, they require expensive cost of equipment and technology for fabricating nanostructures on large-area solar cells. In addition, surface recombination defects induced by etch process will decrease the device performance. Consequently, the nanostructures fabricated by using bottom-up grown methods have been developed [5-7].

Recently, zinc oxide (ZnO) nanostructures have become regarded as suitable for forming efficient antireflection coatings, taking advantage of their good transparency, appropriate refractive index, and ability to be formed as textured coatings by anisotropic growth. Also, ZnO exhibits several favorable material characteristics, such as its abundance, wide direct band gap (3.3 eV), low manufacture cost, non-toxicity, large exciton binding energy, and chemical stability against hydrogen plasma [8,9]. The synthesis of ZnO nanostructures is currently attracting considerable attentions because of their good physical properties. Various ZnO nanostructures have been demonstrated, including nanowires, nanotips, nanotubes, and nanocages [10-13]. This work proposes an effective non-selenized Cu(In,Ga)Se_2_ (CIGS) solar cell with ZnO nanorods on an aluminum (Al)-doped ZnO (AZO) seed layer. This is also of one-stage sputtering process, taking no toxic selenization procedure, low production cost, and no solvent pollution to the environment [14]. It is thereby suitable for large area and mass production. In addition, a simple, low-cost, and environmentally friendly chemical solution-based deposition is developed for growing vertically oriented arrays of hexagonal ZnO nanorods at a low processing temperature. The improvements in the optical reflection properties, the current-voltage (*I*-*V*) characteristics and the external quantum efficiency (EQE) of non-selenized CIGS solar cell are demonstrated with the ZnO nanorod antireflection coatings.

## Methods

CIGS-based photovoltaic devices were fabricated with the structure of soda-lime glass/Mo/CIGS/CdS/ZnO/AZO/Al contact. The p-type CIGS films were deposited by the process described previously [14], employing one-stage deposition cycle and a final heat treatment at 550°C. The cell is completed by a chemical bath deposited CdS buffer layer and a RF-sputtered ZnO/AZO transparent front contact (window layer). Finally, a grid of Al used as a top contact was deposited by sputtering with a contact mask. In order to fabricate the antireflection coating on the top surface of the non-selenized CIGS solar device, ZnO nanostructures were grown by the hydrothermal method. The reaction chemicals were prepared by mixing zinc nitrate hexahydrate (Zn(NO_3_)_2_ · 6H_2_O) and hexamethylene tetramine (C_6_H_12_N_4_, HMT) in aqueous solution. After the solution was stirred for 10 min, bare non-selenized CIGS solar cells were immersed vertically in this solution, and the sealed reaction bottle was heated up to 90°C. The pH value of the chemical solution was adjusted to the desired value from 6.5 to 8 by using 1,3-diaminopropane (DAP, Acros) solution [15]. Field-emission scanning electron microscope (FESEM) images were taken using a JEOL JSM-7401 F instrument (Tokyo, Japan). In order to obtain cross-sectional images, samples were broken mechanically. The surface and cross-sectional microstructures of the films were investigated by FESEM operating at 10 kV. The crystalline structure of the ZnO films was observed by X-ray diffraction (XRD) with an automated Bruker D8 advance X-ray diffractometer (Madison, WI, USA) with CuKα radiation (40 kV and 30 mA) for 2*θ* values of over 20° to 60°. Energy dispersive spectroscopy (EDS) with standardless calibration, using an accelerating voltage of 10 kV, and a dead time of approximately 20%, was performed to determine the composition of deposited ZnO nanorods. Optical transmittance and reflectance were measured at normal incidence in the wavelength range of 400 to 1,200 nm with a Cary 500 UV-visible-near infrared spectrophotometer equipped with an integrated sphere. The current-voltage characteristics of solar cells were measured by a Keitheley 4200 semiconductor analyzer under the irradiation of simulated AM1.5 sunlight with the power density of 100 mW/cm^2^ at 25°C using a temperature controller.

## Results and discussion

**Figure 1:**
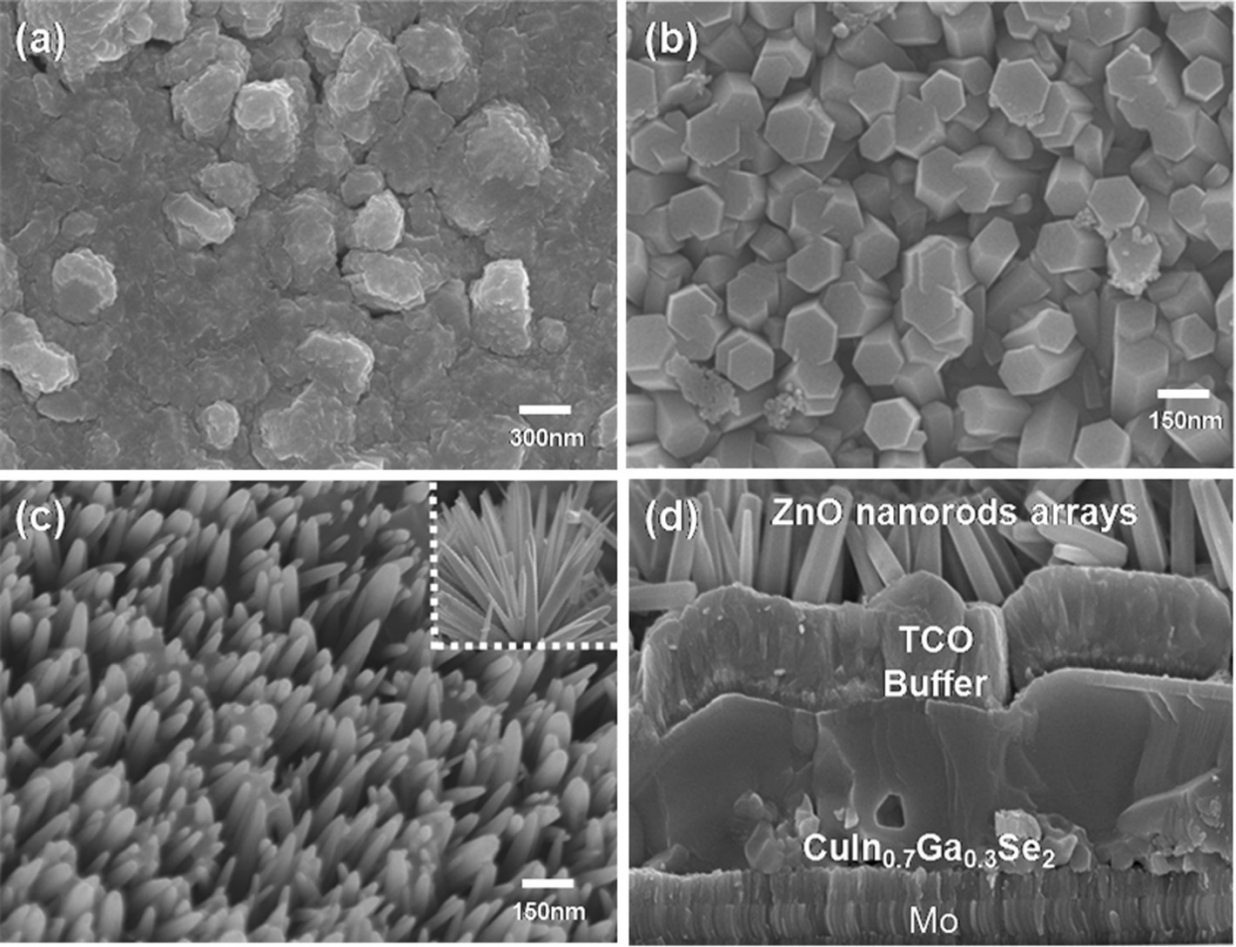
**FESEM images. (a)** AZO film surface of a bare non-selenized CIGS solar cell, **(b)** flat-top and **(c)** tapered ZnO nanorods, and **(d)** cross-sectional FESEM image of CIGS solar cell.

To enhance the efficiency of the non-selenized CIGS solar cells, ZnO nanostructures were synthesized using a two-step method, involving the formation of AZO seed layers and the growth of ZnO nanorods in that order. The surface morphology of a bare non-selenized CIGS solar cell is shown in Figure 1a. The AZO top layer exhibited a bumpy structure with microscale roughness due to the large grain growth of the non-selenized CIGS absorber layer. After the hydrothermal process, two kinds of ZnO nanorods vertically grown on the bumpy AZO films were observed as shown in Figure 1b,c. Variations in the growth conditions of nanorod array growth conditions strongly influenced the nanoscale morphology of the textured ZnO antireflection coatings, as shown by the FESEM images (Figure 1). In this work, at a growth temperature of 90°C, the tips of the ZnO nanorods changed from a flat top (Figure 1b) to a tapered shape (Figure 1c) with the an addition of DAP into the growth solution. Generally, in order to achieve an efficient solar cell with antireflection structures for maximum transmittance and minimum reflectance without the occurrence of diffraction and scattering loss, the following conditions should be conformed [16-19]:

1. Conical region of ZnO nanorod must have a height (h) equal to at least 40% of the longest operational wavelength.

2. Center-to-center spacing of ZnO nanorod must be less than the shortest operational wavelength divided by the refractive index (n) of the material.

It was recognized that the size and the shape of nanorods grown on the non-selenized CIGS solar cell satisfy the theoretical requirements for the efficient antireflection coating fabrication.

**Figure 2:**
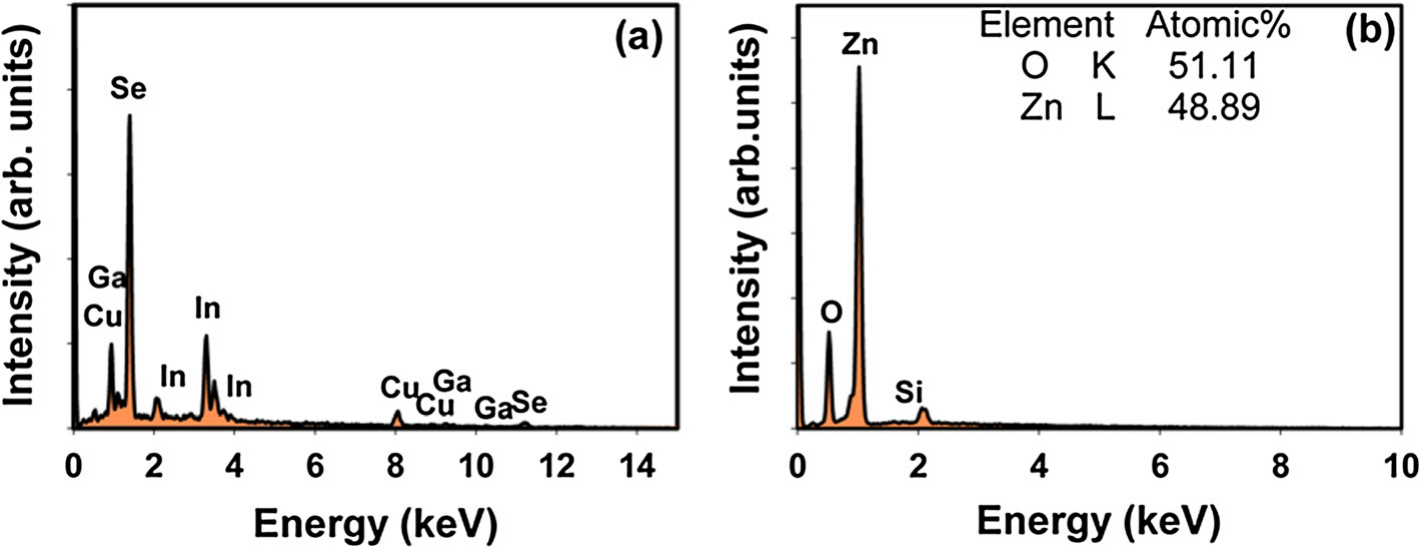
EDS composition analysis of CIGS thin-film (a) and ZnO nanorods (b).

EDS with standardless calibration was used to determine the composition of deposited CIGS film by using an accelerating voltage of 15 kV and a dead time of approximately 20%. The EDS composition analysis shows that the CIGS film, shown in Figure 2a, is composed of Cu 24.33%, In 16.78%, Ga 7.71%, and Se 51.18% (at.%). The film composition was designed to include Cu-poor and In-rich compositions [approximately Ga/(Ga + In) = 0.31, In/(Ga + In) = 0.68, and Cu/(Ga + In) = 0.99]. The band gap energy of Cu(In_1−*x*_Ga_*x*_)Se_2_ follows a parabolic function of *x*, and its behavior can be expressed as Eg(*x*) = (1 − *x*) Eg(CIS) + *x*Eg (CGS) − *bx*(1 − *x*), where *b* is the bowing parameter with a value of 0.15 eV for Cu(In_1−*x*_Ga_*x*_)Se_2_ thin films. Eg(CIS) = 1 eV and Eg(CGS) = 1.67 eV are the band gaps of CuInSe_2_ and CuGaSe_2_, respectively [20]. All CIGS layers were of comparable thickness. The energy band gap of CIGS films is 1.17 eV with Ga/ (Ga + In) = 0.3 is suitable for acting as absorbers. To confirm the composition of the proposed ZnO nanorods, EDS spectra coupled to FESEM is recorded and analyzed. Figure 2b shows a typical EDS spectrum generated using FESEM, which demonstrates that zinc and oxygen were detected elements and minor silicon. The presence of silicon could be explained by soda-lime glass which is composed of about 75% silica (SiO_2_) plus sodium oxide from soda ash and lime.

**Figure 3:**
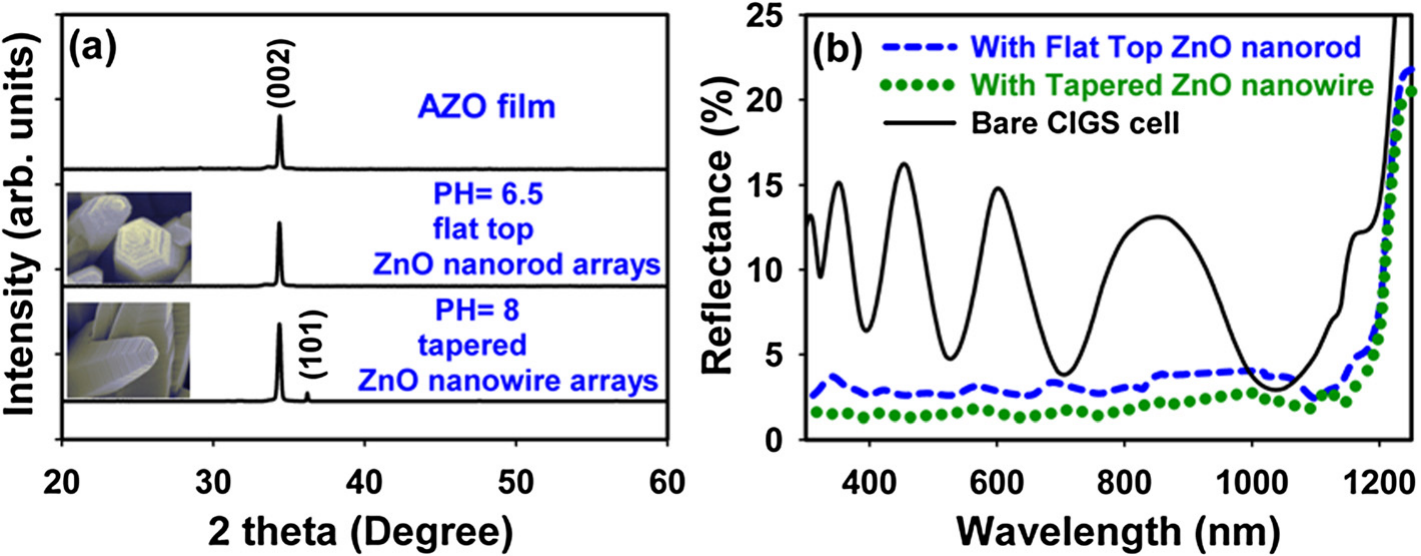
**XRD spectra (a) and wavelength-dependent reflectance (b). (a)** XRD spectra of AZO film surface and antireflection coatings of the flat-top ZnO nanorods and the tapered ZnO nanorods. **(b)** Wavelength-dependent reflectance of non-selenized CIGS solar cell before (black line) and after (blue and green lines) deposition of antireflection coating of nanorods.

Figure 3a presents the crystal structure and preferential orientation of ZnO nanorods on AZO/glass formed at the pH values of 6.5 and 8, respectively. XRD pattern of the prepared ZnO was recorded using an automated Bruker D8 with CuKα radiation. The XRD spectra of ZnO nanorods include a dominant peak at 34.4°, associated with the (002) plane of ZnO crystals, as well as a weak (101) peak. All ZnO arrays yielded diffraction peaks of pure ZnO crystals with a hexagonal structure, suggesting that the films were oriented along the *c*-axis perpendicular to the AZO window layer because the (002) reflection was much greater than the usual (101) maximum reflection. To evaluate the performance of the antireflective coating on the non-selenized CIGS solar cell, absolute hemispherical reflectance measurements with an integrating sphere were made over the visible to near-IR spectral range, as shown in Figure 3b showing the average reflectance of a bare CIGS solar cell, which was measured to be 8.6% for the UV-visible wavelength range. Comparatively, the average reflectance of ZnO-covered CIGS solar cells with antireflection coating patterns of flat top and tapered ZnO nanostructures were measured to be 3.2% and 2.1%, respectively. The reflectance spectra of the non-selenized CIGS solar cells with ZnO nanorod antireflective coating were clearly lower than those of the cells without it over wavelengths ranging from the ultraviolet to the near-infrared. The reflectance spectra of the non-selenized CIGS cell without an antireflective layer exhibited interference fringes. In contrast, the spectra of the ZnO nanorod-coated CIGS cell revealed significantly low reflectance, and the interference fringes were not observed at visible wavelength. The suppression of the optical reflectance of wavelengths from 400 to 1,000 nm was close to constant. It can be attributed to the reduction in reflection and the enhancement of photon absorption by the coating layer of ZnO nanorods. This suppression is caused by the moth-eye effect that originates from a graded refractive index in the textured ZnO nanorod-coated antireflective layer. These results reveal that the non-selenization CIGS cell device with ZnO-nanostructure coatings can absorb more photons and converted them into electrical current, owing to its excellent light-trapping ability [21]. The synthesis of ZnO nanorods in aqueous solution was a simple fabrication method of subwavelength-textured coatings to suppress the reflection of visible to near-infrared wavelengths. The method differs from other complicated methods, such as the electronbeam, followed by etching.

**Figure 4:**
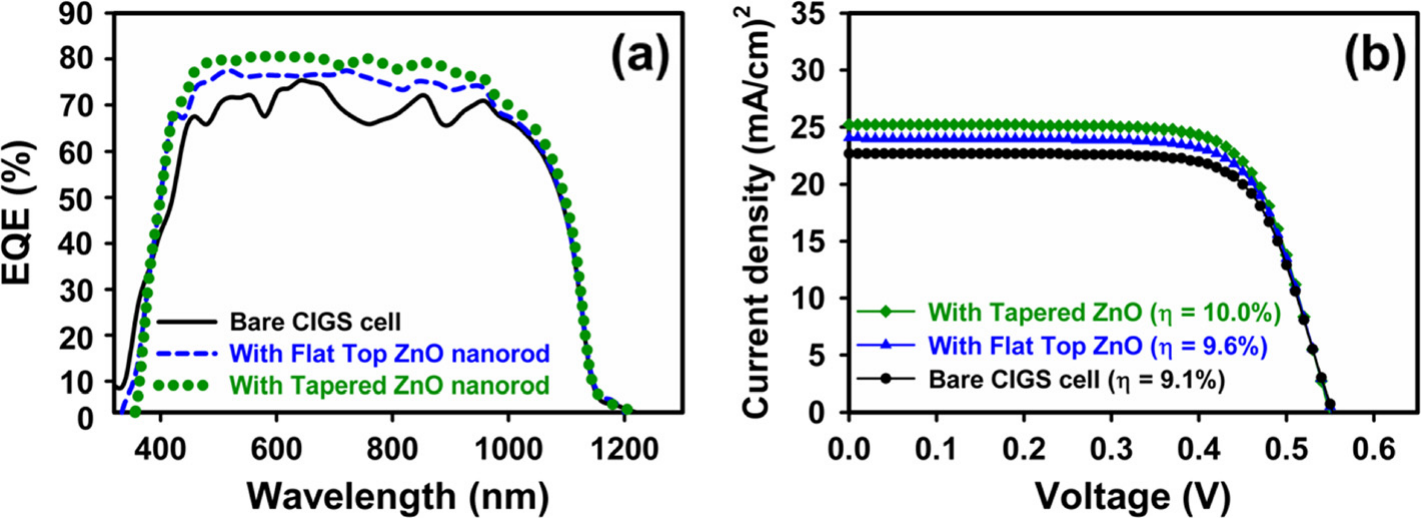
**External quantum efficiency (a) and current-voltage characteristics (b) of solar cells. (a)** Solar cell before (black line) and after (blue and green lines) deposition of antireflection coating of nanorods. **(b)** Bare non-selenized CIGS solar cell and flat-top/tapered ZnO nanorod antireflection-coated non-selenized CIGS solar cells.

The EQE of the CIGS solar devices was also measured to evaluate the effect of ZnO nanorod coating layer on performance improvement. Figure 4a compares the EQE data for the non-selenization CIGS devices with and without the ZnO nanorod antireflection coating layer. The CIGS cell with ZnO nanorods had excellent quantum efficiency at wavelengths ranging from 450 to 950 nm, owing to the low optical reflectance of the ZnO nanorods. The quantum efficiency of non-selenization CIGS cell with ZnO nanostructure drops off at a high energy of approximately around 320 nm -a lower energy than that without the antireflection coatings. This phenomenon is caused by the fact that the optical band gap energy of ZnO is lower than that of the high band gap material, of AZO layer [22], owing to the Burnstein-Moss bandgap effect. Figure 4b plots the photocurrent versus applied voltage (*J*-*V*) curve for the CIGS solar cells with and without the ZnO antireflection coatings under AM1.5 illumination. The CIGS solar cell with tapered ZnO nanorods reaches an efficiency as high as 10% to 11%. The cell conversion efficiency is 9.1% with an open-circuit voltage of 0.55 V, a short current density of 22.7 mA/cm^2^, and a fill factor (FF) of 72.3%. Based on the *J*-*V* curves, the increase of the short-circuit current is believed to be related to the decrease in reflectance that is caused by the ZnO nanostructure antireflective coating layer. The gain in photocurrent due to the antireflective effect could be given by the previous work [23]. In this study, the comparative advantages that are provided by the ZnO nanostructures on non-selenized CIGS solar cells are indicated by the extra gain in the photocurrent *G*_p_ (*G*_p_ ≡ Δ*J*_sc_/*J*_sc_), 11%, for the tapered ZnO nanorods. The tapered ZnO nanorod coating ultimately increased the efficiency of non-selenized CIGS solar cells by 9.8% from 9.1% to 10%. There are obvious improvements in photocurrent and efficiency enhancement. These are mainly caused by both the reduction of light reflectance and surface recombination centers by the window layer [24-27].

Based on the results of flat-top and tapered ZnO antireflection coatings, we also observed that the light conversion efficiency was improved by over 10% and that photocurrent was increased by more than 11%. They can be attributed to the enhanced light absorption caused by the multiple photon scattering phenomena associated with the nanorod arrays. According to the weighted reflectance *R*_w_[23] with both the internal spectral response of the solar cell and the AM1.5 solar spectrum, we found that decreasing the nanorod tip diameter to 50 nm improved the *R*_w_ from 13.5% to 12.6% in the letter. According to the effective medium theory [28], the effective refractive index increases with the filling factor. The filling factors at the air-ZnO nanorod array interface are statistically estimated to be 17.21% and 12.47% for flat-top and tapered ZnO, respectively. Consequently, tapered ZnO nanorod arrays have the lowest effective refractive index at the interface.

Table 1 lists the electrical parameters for all CIGS devices with tapered ZnO nanorod coating. Several concentrations of DAP were also added to control the tip diameter of tapered nanorods. Six as-fabricated CIGS solar cells prepared from the same batch presented the conversion efficiency and current density of approximate 9.1% and 22.7 mA/cm^2^, respectively. After covering with 20-nm-diameter ZnO nanorod on the top of solar devices, the efficiency and current density were improved to 11.1% and 29.5 mA/cm^2^, respectively. This photocurrent increase, related to the increase of photon excitation in the CIGS absorber, enhanced photovoltaic efficiency after introducing ZnO nanorod antireflection coatings. However, the performances of CIGS solar cells were not further enhanced according to further weighted reflectance reduction in other samples. The tapered ZnO nanorod tip diameter has been varied to find out the optimum diameter for the conventional non-selenized CIGS structure with ZnO nanorod as the antireflection coatings. It has been found that the efficiency of the solar cell is increasing with the decreasing of the tip diameter of the ZnO nanorod, but with a much slower rate under 30 nm. The optimum diameter for ZnO nanorod would be around 20 to 30 nm.

**Table 1:** Photovoltaic performance of non-selenized CIGS solar cells with different conditions of ZnO nanorod antireflection coating

**Device**	**Tapered ZnO nanorods**	**Electrical properties**					
**ID**	**(diameter, nm)**	**Voc (mV)**	**FF (%)**	**Jsc (mA/cm**^**2**^**)**	***η *** **(%)**	**Improvement (*η*, %)**	**Rw (%)**
1	-	553	72.3	22.7	9.1		25.1
2	50	551	72.2	25.2	10.0	+9.8	12.6
3	40	552	72.2	26.9	10.7	+17.5	9.6
4	30	552	70.1	28.5	11.0	+20.8	9.1
5	20	553	68.4	29.4	11.1	+21.9	9.1
6	15	553	68.4	29.5	11.1	+21.9	9.0

## Conclusions

In summary, the effects of ZnO nanorods as a subwavelength-textured antireflection coating on non-selenized CIGS thin-film solar cell have been demonstrated in this work. Based on the moth-eye effect, the reflection on the surface of CIGS solar cell covered with nanostructured ZnO layer can be effectively eliminated. The surface morphology of ZnO nanostructures also played a critical role in the reduction of the reflection. With the coating of branched tapered ZnO nanorods, the average reflectance of the non-selenized CIGS solar cell decreased the magnitude by three times, and the energy conversion efficiency increased by 20%. The aqueous-grown ZnO nanostructures also can be fabricated with a large-area coating process at a temperature less than 90°C. It thereby would have a great potential for further application to flexible solar cell technology.

## Competing interests

The authors declare that they have no competing interests.

## Authors’ contributions

B-TJ wrote the paper and did the experiment. P-TL guided the experiment. M-CW participated in the design of the study and the instructions of the calculations. All authors read and approved the final manuscript.
